# GA-GBLUP: leveraging the genetic algorithm to improve the predictability of genomic selection

**DOI:** 10.1093/bib/bbae385

**Published:** 2024-08-05

**Authors:** Yang Xu, Yuxiang Zhang, Yanru Cui, Kai Zhou, Guangning Yu, Wenyan Yang, Xin Wang, Furong Li, Xiusheng Guan, Xuecai Zhang, Zefeng Yang, Shizhong Xu, Chenwu Xu

**Affiliations:** Key Laboratory of Plant Functional Genomics of the Ministry of Education/Jiangsu Key Laboratory of Crop Genomics and Molecular Breeding/Jiangsu Co-Innovation Center for Modern Production Technology of Grain Crops, College of Agriculture, Yangzhou University, Yangzhou, Jiangsu 225009, China; Key Laboratory of Plant Functional Genomics of the Ministry of Education/Jiangsu Key Laboratory of Crop Genomics and Molecular Breeding/Jiangsu Co-Innovation Center for Modern Production Technology of Grain Crops, College of Agriculture, Yangzhou University, Yangzhou, Jiangsu 225009, China; College of Agronomy, Hebei Agricultural University, Baoding, Hebei 071001, China; Key Laboratory of Plant Functional Genomics of the Ministry of Education/Jiangsu Key Laboratory of Crop Genomics and Molecular Breeding/Jiangsu Co-Innovation Center for Modern Production Technology of Grain Crops, College of Agriculture, Yangzhou University, Yangzhou, Jiangsu 225009, China; Key Laboratory of Plant Functional Genomics of the Ministry of Education/Jiangsu Key Laboratory of Crop Genomics and Molecular Breeding/Jiangsu Co-Innovation Center for Modern Production Technology of Grain Crops, College of Agriculture, Yangzhou University, Yangzhou, Jiangsu 225009, China; Key Laboratory of Plant Functional Genomics of the Ministry of Education/Jiangsu Key Laboratory of Crop Genomics and Molecular Breeding/Jiangsu Co-Innovation Center for Modern Production Technology of Grain Crops, College of Agriculture, Yangzhou University, Yangzhou, Jiangsu 225009, China; Key Laboratory of Plant Functional Genomics of the Ministry of Education/Jiangsu Key Laboratory of Crop Genomics and Molecular Breeding/Jiangsu Co-Innovation Center for Modern Production Technology of Grain Crops, College of Agriculture, Yangzhou University, Yangzhou, Jiangsu 225009, China; Key Laboratory of Plant Functional Genomics of the Ministry of Education/Jiangsu Key Laboratory of Crop Genomics and Molecular Breeding/Jiangsu Co-Innovation Center for Modern Production Technology of Grain Crops, College of Agriculture, Yangzhou University, Yangzhou, Jiangsu 225009, China; Key Laboratory of Plant Functional Genomics of the Ministry of Education/Jiangsu Key Laboratory of Crop Genomics and Molecular Breeding/Jiangsu Co-Innovation Center for Modern Production Technology of Grain Crops, College of Agriculture, Yangzhou University, Yangzhou, Jiangsu 225009, China; Global Maize Program, International Maize and Wheat Improvement Centre, Texcoco 56237, Mexico; Key Laboratory of Plant Functional Genomics of the Ministry of Education/Jiangsu Key Laboratory of Crop Genomics and Molecular Breeding/Jiangsu Co-Innovation Center for Modern Production Technology of Grain Crops, College of Agriculture, Yangzhou University, Yangzhou, Jiangsu 225009, China; Department of Botany and Plant Sciences, University of California, Riverside, CA 92521, United States; Key Laboratory of Plant Functional Genomics of the Ministry of Education/Jiangsu Key Laboratory of Crop Genomics and Molecular Breeding/Jiangsu Co-Innovation Center for Modern Production Technology of Grain Crops, College of Agriculture, Yangzhou University, Yangzhou, Jiangsu 225009, China

**Keywords:** genetic algorithm, genomic best linear unbiased prediction, genomic selection, feature selection

## Abstract

Genomic selection (GS) has emerged as an effective technology to accelerate crop hybrid breeding by enabling early selection prior to phenotype collection. Genomic best linear unbiased prediction (GBLUP) is a robust method that has been routinely used in GS breeding programs. However, GBLUP assumes that markers contribute equally to the total genetic variance, which may not be the case. In this study, we developed a novel GS method called GA-GBLUP that leverages the genetic algorithm (GA) to select markers related to the target trait. We defined four fitness functions for optimization, including AIC, BIC, R^2^, and HAT, to improve the predictability and bin adjacent markers based on the principle of linkage disequilibrium to reduce model dimension. The results demonstrate that the GA-GBLUP model, equipped with R^2^ and HAT fitness function, produces much higher predictability than GBLUP for most traits in rice and maize datasets, particularly for traits with low heritability. Moreover, we have developed a user-friendly R package, GAGBLUP, for GS, and the package is freely available on CRAN (https://CRAN.R-project.org/package=GAGBLUP).

## Introduction

Genomic selection (GS), first proposed by Meuwissen in 2001 [1], aims to predict genomic estimated breeding value (GEBV) utilizing genome-wide markers. GS has revolutionized modern animal breeding via shortening generation intervals and increasing genetic gain. GS has become a widely used tool in dairy cattle breeding worldwide, doubling the rate of genetic gain for yield and yield related traits [2]. Inspired by the huge success in animal breeding, GS has been introduced to crop breeding in several areas including pure line selection and hybrid prediction [3, 4]. In the context of crop hybrid breeding, GS proves to be more efficient as hybrid genotypes can be inferred from their parents, eliminating the need for new sequencing and significantly lowering the costs. For example, in rice, Xu et al. used 278 hybrids derived from 210 inbred lines as a training sample to predict all 21 945 potential crosses and concluded that if the top 100 crosses were selected, the yield would be increased by 16% [5]. In maize, Li et al. predicted all 6328 potential crosses based on 490 hybrids and found that the selection of the top 44 crosses would lead to a 6% growth in grain yield (GY) compared to the hybrid breed of Zhengdan 958 [6].

Numerous statistical methods have been developed for GS, such as genomic best linear unbiased prediction (GBLUP), various Bayesian methods, and machine learning methods. Although no single method is universally best for all datasets, GBLUP stands out for its robustness and computationally efficiency [7, 8], making it a routinely used method in GS breeding programs. However, GBLUP assumes that all markers follow a common normal distribution with the same genetic variance, which is more applicable to polygenic traits with a large number of minor effects rather than traits controlled by a few major genes [9, 10]. To improve the predictability of GBLUP, several studies have suggested a strategy that includes significant markers identified in genome-wide association studies (GWAS) as fixed or random effects in GBLUP [8, 11, 12]. However, the effectiveness of the joint GS-GWAS analysis largely hinges on the genetic architecture of target traits. For instance, a simulation study in maize and sorghum demonstrated that incorporating the peak markers from GWAS conducted on the training population increased the predictability for only 60 out of 216 simulated traits compared to the BLUP model [12]. Therefore, it is natural to develop improved methods within the GBLUP framework for traits with diverse genetic architectures.

Genetic algorithm (GA), first conceptualized by Holland, is an optimization algorithm used to search for a parameter set that maximizes a target function (referred to as fitness) drawing inspiration from the Darwinian theory of ‘survival of the fittest’ [13]. As a population-based metaheuristic algorithm, GA utilizes multiple candidate solutions during the search process to find an optimal solution [14]. It mimics the slow process of evolution for a population of living organisms to adapt to a particular environment via changes in the genetic composition of the population. The evolutionary process in GA starts with an initial population of individuals. The individuals in the population, akin to the chromosomes of biological organisms, are encoded by bit strings with binary values. It then goes through iterations termed generations, during which individuals undergo a series of genetic operations, including selection, crossover, and mutation. The selection operator selects individuals based on their fitness values to reproduce and generate offspring for the next generation. The crossover operator exchanges segments of two selected individuals to produce new offspring. The mutation operator introduces new random variation into the population, promoting genetic diversity and avoiding premature convergence on suboptimal solutions. Due to its evolutionary nature, GA has a higher probability of finding the global optimum (with the highest fitness) compared to many other algorithms that may become trapped in local optima. GA proves to be particularly effective in scenarios with vast solution spaces, making it well suited for identifying the optimal subset of trait-specific markers. Despite its potential, GA has not yet been utilized for such a purpose.

In this study, we adapted the GA to GS and developed a new GS method called GA-GBLUP aiming to target traits with a diverse range of genetic architectures. We also defined four different fitness functions (AIC, BIC, R^2^, and HAT) and provided flexible choices for users to consider in their own GS programs. The computational efficiency of GA was further improved by binning (combining) neighboring markers based on the principle of linkage disequilibrium (LD). To demonstrate the effectiveness of the proposed method, we applied GA-GBLUP to a hybrid rice population and a hybrid maize population. Finally, we developed an R package (R/GAGBLUP) to help breeders perform GS using the new method.

## Methods

### The hybrid rice population

The rice population consists of 278 hybrids generated by random mating among 210 recombinant inbred lines (RILs). The 210 RILs were derived by single-seed descent from a cross between Zhenshan 97 and Minghui 63 [15, 16]. We analyzed four traits including grain yield per plant (YIELD), number of tillers per plant (TILLER), number of grains per panicle (GRAIN), and 1000-grain weight (KGW). The traits were measured on the experimental farm of Huazhong Agricultural University in 1998 and 1999. The field trials were conducted with a randomized complete block design with two replicates per year [16]. In each replicate, eight plants were sampled from each hybrid, and the average phenotypic values were used as the phenotypic values for analysis. The genotypes of the 210 RILs were represented by 1619 recombinant bins inferred from 270 820 high-quality SNPs of the rice genome, and the genotypes of the hybrids were deduced from the bin genotypes of their parents [17]. For ease of reference, the abbreviations used throughout the paper are listed in Table 1.

**Table 1:** List of abbreviations

**Classification**	**Abbreviation**	**Definition**	**Units**
Traits	YIELD	Grain yield per plant	g
	TILLER	Number of tillers per plant	–
	GRAIN	Number of grains per panicle	–
	KGW	1000-grain weight	g
	PH	Plant height	cm
	EH	Ear height	cm
	EW	Ear weight	g
	EGW	Ear grain weight	g
	GY	Grain yield	t/ha
	GDMC	Grain dry matter content	%
Methods	GS	Genomic selection	–
	BLUP	Best linear unbiased prediction	–
	GBLUP	Genomic best linear unbiased prediction	–
	GA	Genetic algorithm	–
	GEBV	Genomic estimated breeding value	–
	GWAS	Genome-wide association studies	–
	LASSO	Least absolute shrinkage and selection operator	–
	LD	Linkage disequilibrium	–
	PCA	Principal component analysis	–
	PC	Principal component	–
	PCA95	Principal components explaining 95% of the total variance of genotypic data	–
	REML	Restricted maximum likelihood	–
	CV	Cross-validation	–
	LOOCV	Leave-one-out cross-validation	–

### The hybrid maize population

The maize305 dataset, consisting of 305 hybrids, was constructed using a sparse partial diallel crossing design involving 149 parental lines. The phenotypic data of plant height (PH), ear height (EH), ear weight (EW), and ear grain weight (EGW) of the 305 hybrids were collected from the experimental farms in Yangzhou and Tai’an during the maize growing seasons of 2017 and 2018 [18, 19]. The field trials were conducted with a randomized block design with two replicates in each environment. For each replicate, each line was planted in a single-row plot with 13 plants (3.0 m in length and 0.5 m between rows), and five uniform plants per plot were selected for phenotyping. The best linear unbiased prediction (BLUP) values for each hybrid were calculated with R/lme4 and were used as the ‘phenotypes’ for the GS study. Each inbred parent was genotyped using a 40 K maize liquid array developed by Molbreeding Biotechnology Company (Shijiazhuang, Hebei, China). After quality control via removing SNPs with a missing rate greater than 10% and markers with minor allele frequencies less than 5%, we obtained 41 806 SNPs. SNP genotypes of the hybrids were deduced from the genotypes of their inbred parents.

### Genotype coding

Let $M=\left\{{M}_{jk}\right\}$ and $F=\left\{{F}_{jk}\right\}$ be $n\times m$ genotype matrices for the male and female parents of the corresponding hybrids, respectively. The numerical code for individual *j*$\left(j=1,2,...,n\right)$ at marker *k*$\left(k=1,2,...,m\right)$ is defined as ${M}_{jk}={F}_{jk}=1$ for the homozygote of the major allele ${A}_1$, ${M}_{jk}={F}_{jk}=0$ for the heterozygote ${A}_1{A}_2$, and ${M}_{jk}={F}_{jk}=-1$ for the homozygote of the minor allele ${A}_2$. The genotype of the hybrid is defined as ${H}_{jk}=\frac{1}{2}\left({M}_{jk}+{F}_{jk}\right)$. Note that the parents are not 100% inbred for all markers. This explains why the parent’s genotypes may be coded as 0. The coded genotype of a hybrid may take values of −1, −0.5, 0, 0.5, and 1.

### Dimension reduction

To reduce the dimension of the genotype matrix, we combined neighboring SNPs into bins. The term ‘bin’ here refers to a block of physically linked SNPs exhibiting high LD [20], as opposed to the traditional definition where a bin is a chromosome block without recombination in a segregating population. Two steps are needed to create bin genotypes for a population with numeric genotypes: standardizing the genotype matrix and combining adjacent SNPs into bins according to some predetermined criterion. First, let $G$ be an *n* × *r* standardized genotype matrix, where each column has a mean 0 and a standard deviation 1. Let ${G}_{pq}$ be the *p*th marker for all *n* individuals in the *q*th bin, where $p=1,2,...,{r}_q$ and ${r}_q$ is the number of markers within the *q*th bin. Adjacent markers need to be combined to create bins. Let ${B}_q$ be the average value of all ${r}_q$ markers in the *q*th bin:


(1) \begin{equation*} {B}_q=\frac{1}{r_q}\sum \limits_{p=1}^{r_q}{G}_{pq} \end{equation*}

The actual variance of bin *q* is defined as


(2) \begin{equation*} \operatorname{var}\left({B}_q\right)=\frac{1}{r_q^2}\left[{r}_q+2\sum \limits_{p=1}^{r_q-1}\sum \limits_{l=p+1}^{r_q}{d}_{pl}\right] \end{equation*}

where ${d}_{pl}$ is the LD parameter (correlation coefficient) between markers *p* and *l* within the *q*th bin. We can see that $\operatorname{var}\left({B}_q\right)$ varies between $1/{r}_q$ and 1. Therefore, the number of SNPs in bin *q* (${r}_q$) is determined by


(3) \begin{equation*} \left[\operatorname{var}\left({B}_q\right)>v\right]\wedge \left[\operatorname{var}\left({B}_{q+1}\right)<v\right] \end{equation*}

where *v* is a hyperparameter of the binning algorithm ranging from 0 to 1. We can adjust *v* to control the number of bins, the smaller the value of *v*, the fewer the number of bins. We also used principal component analysis (PCA) to reduce the genotype dimension. The PCA was performed using the R/prcomp function to extract the top principal components (PCs) with a cumulative explanatory variance of 95%. This PCA procedure is referred to as PCA95 hereafter.

### The GA-GBLUP method

Let *y* be an $n\times 1$ vector for the phenotypic values of a target trait. The GA-GBLUP model is formulated as


(4) \begin{equation*} y= X\beta +\sum \limits_{k=1}^m{\delta}_k{Z}_k{\gamma}_k+\varepsilon \end{equation*}

where $X$ is an $n\times q$ design matrix for the fixed effects, $\beta$ is a $q\times 1$ vector of the fixed effects, ${\delta}_k$ is an indicator variable for marker *k*, with ${\delta}_k=1$ indicating the inclusion of marker *k* in the model, while ${\delta}_k=0$ indicates the exclusion of marker *k* from the model. Vector $\delta =[{\delta}_1\kern0.5em {\delta}_2\kern0.5em \begin{array}{cc@{}}...& {\delta}_m\end{array}]$ is considered the parameter vector (bit strings) with a binary value at each locus. For example, if $\delta =[\begin{array}{@{}cccccc@{}}1& 1& 0& 1& 0& 0\end{array}]$ is a candidate solution, markers 1, 2, and 4 are included in the model while markers 3, 5, and 6 are excluded. ${Z}_k$ is an $n\times 1$ vector for the genotype indicator variable of all *n* individuals for marker *k*, $\gamma =\left\{{\gamma}_{\mathrm{k}}\right\}$ is a vector of random effects for all markers with an assumed $N\left(0,{\sigma}_{\gamma}^2/m\right)$ distribution and $\varepsilon$ is a vector of residual errors with $N\left(0,I{\sigma}^2\right)$ distribution. The expectation of *y* is $E(y)= X\beta$ and the variance-covariance matrix is $\operatorname{var}(y)=V=A{\sigma}_{\gamma}^2+I{\sigma}^2$, where *A* is an $n\times n$ kinship matrix derived from selected makers. The parameters $\left\{\beta, \kern0.33em {\sigma}_{\gamma}^2,\kern0.33em {\sigma}^2\right\}$ can be estimated using the restricted maximum likelihood (REML) method described in detail in our previous study [5]. If the number of markers is large, it is nearly impossible to evaluate all possible compositions of $\delta$. However, GA allows the population of $\delta$ to evolve so that the optimal set of $\delta$ that maximizes the fitness of the model can be slowly approached. The specific implementation process is described as follows:

1. Initialization

We first initialize $N=100$ times for vector $\delta$, denoted by ${\delta}^{(i)}$ for the *i*th replication for $i=1,2,...,N$, where *N* is the population size. The initial value of ${\delta}^{(i)}$ is assigned values sampled from a Bernoulli distribution with probability $\pi =0.01$ for each locus.

1. Selection

After initialization, we select individuals with the highest fitness value according to a predetermined fitness function. The fitness function is the core of GA, which determines whether an individual is selected to breed or not [21]. Four different fitness functions are proposed, and they are described as follows:

(1) Akaike information criterion (AIC). AIC is a standard measurement of the model optimality [22]. It is based on the concept of entropy and balances the model simplicity and goodness of fit. The AIC is defined as


(5) \begin{equation*} \mathrm{AIC}=2k-2\ln (L) \end{equation*}

where $k$ is the number of parameters being estimated, $L$ is the likelihood function of the model evaluated at the maximum likelihood estimates of the parameters. The smaller the AIC, the better the model.

(2) Bayesian information criterion (BIC). BIC takes into account the sample size in calculating the information criterion [23]. The BIC is defined as


(6) \begin{equation*} \mathrm{BIC}=k\ln (n)-2\ln (L) \end{equation*}

where $k$ is the number of parameters being estimated, $n$ is the sample size and $L$ is the likelihood function of the model. The smaller the BIC, the better the model. AIC and BIC are the same when $\ln (n)=2$, which is equivalent to $n=\exp (2)=7.389$. Since the sample size of any data analysis is expected to be greater than 8, the BIC has a greater penalty than the AIC.

(3) The R-squared (R^2^). The R^2^ represents the goodness of fit of a model, which is defined as


(7) \begin{equation*} {\mathrm{R}}^2=1-\mathrm{S}{\mathrm{S}}_{\mathrm{E}}/\mathrm{S}{\mathrm{S}}_{\mathrm{T}} \end{equation*}

where SS_T_ is the total sum of squares of the phenotypic values, and SS_E_ is the residual sum of squares.

(4) The HAT fitness function (HAT). Following the HAT method described by Xu [24], we defined the hat matrix as


(8) \begin{equation*} H={H}^F+{H}^R\left(I-{H}^F\right) \end{equation*}

where


(9) \begin{equation*} {H}^F=X{\left({X}^T{V}^{-1}X\right)}^{-1}{X}^T{V}^{-1} \end{equation*}

and


(10) \begin{equation*} {H}^R={\hat{\sigma}}_{\gamma}^2\mathrm{A}{\left(\mathrm{A}{\hat{\sigma}}_{\gamma}^2+I{\hat{\sigma}}^2\right)}^{-1} \end{equation*}

Let the fitted phenotypic value be


(11) \begin{equation*} \hat{y}=X\hat{\beta}+{\hat{\sigma}}_{\gamma}^2\mathrm{A}{\left(\mathrm{A}{\hat{\sigma}}_{\gamma}^2+I{\hat{\sigma}}^2\right)}^{-1}\left(y-X\hat{\beta}\right)= Hy \end{equation*}

The predicted residual error sum of squares of the mixed model is defined as


(12) \begin{equation*} \mathrm{PRESS}=\sum \limits_{j=1}^n{\left({y}_j-{\hat{y}}_j\right)}^2/{\left(1-{h}_{jj}\right)}^2 \end{equation*}

where ${h}_{jj}$ is the *j*th diagonal element of matrix *H.* The HAT value is defined as


(13) \begin{equation*} \mathrm{HAT}=1-\mathrm{PRESS}/\mathrm{S}{\mathrm{S}}_{\mathrm{T}} \end{equation*}

where


(14) \begin{equation*} \mathrm{S}{\mathrm{S}}_{\mathrm{T}}=\sum \limits_{j=1}^n{\left({y}_j-\overline{y}\right)}^2 \end{equation*}

is the total sum of squares. Once the fitness values of the 100 candidates are obtained for the desired fitness function, the top 5% candidates are selected for subsequent biological-inspired operations.

1. Mutation

**Figure 1:**
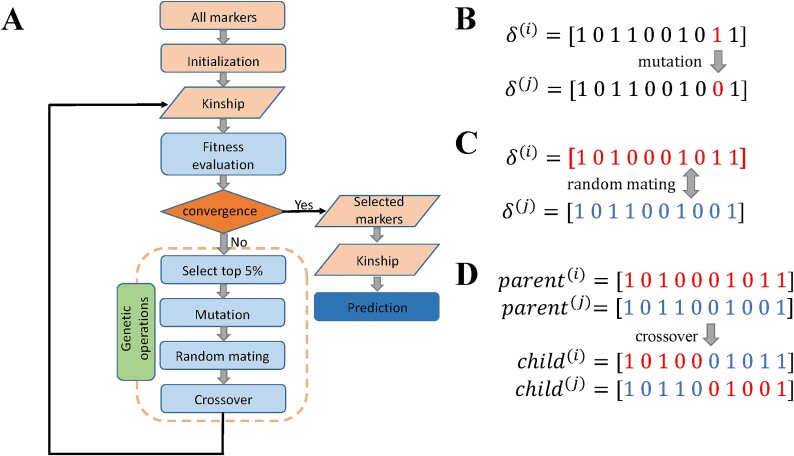
Illustration of the workflow of GA-GBLUP. (A) Flowchart of GA-GBLUP. First, all original markers are input into the algorithm and then a randomly selected subset are used to initiate the algorithm. Next, a kinship matrix is built upon selected markers and fitness values are calculated. Genetic operations are then performed to optimize the solution and recalculate fitness values. This iterative process continues until convergence is achieved. Finally, the selected markers are used for the final prediction. (B) Mutation operation, where the ninth locus mutates from 1 to 0. (C) Random mating operation, where two chromosomes are randomly mated. (D) Crossover operation, a random crossover happens between two mated chromosomes and two progeny chromosomes are created.

Given a mutation rate $\mu =0.1$ per locus, we allow each locus of the selected top 5% chromosomes to mutate, i.e. $1\to 0$ or $0\to 1$. For example, in Fig. 1(B), the ninth locus mutates from 1 to 0, indicating that the ninth locus is excluded from the model.

1. Random mating

We randomly select a pair of individuals to mate. Each mating pair generates two progenies, such a random mating process continues until *N* = 100 progenies are generated. In this case, *N*/2 = 50 random mating pairs need to be created. For example, the first random mating pair may happen between the *i*th and the *j*th selected parents (Fig. 1C).

1. Crossover

Once the mating pairs are selected, crossover occurs between the mated chromosomes. Different forms of crossover, such as single-point and uniform crossovers are used to generate new offspring from parent solutions. In GA-GBLUP, the single point crossover is chosen due to its simplicity and efficiency in maintaining beneficial combinations of loci [25]. In this process, a random crossover point is selected based on a Bernoulli distribution. Segments beyond this point swap with each other, leading to changes in their offspring [14]. For example, if a crossover happens between the 5th and 6th locus, segments of the *i*th (in red) and the *j*th (in blue) parents exchange, resulting in the formation of two children (Fig. 1D).

Finally, two progenies are generated for each mating pair until a total of *N* progenies are produced. The fitness of each progeny is then evaluated, and the top 5% progenies are selected again to generate individuals of the next generation. This iterative process continues until there is no obvious improvement in the fitness function for 2000 consecutive generations. Once the $\delta$ vector with the highest fitness value is obtained by GA, trait-specific markers can be selected to construct a trait-specific kinship matrix (Fig. 1A). We have released an R package GAGBLUP specifically for GA-GBLUP, and this package is available at https://CRAN.R-project.org/package=GAGBLUP.

### Predictability drawn from cross-validation

We adopted the 10-fold cross-validation (CV) scheme to evaluate the performance of GBLUP and GA-GBLUP. Each dataset was partitioned into 10 equal-sized parts with 9 parts used for training and the remaining part for testing. The testing sample does not contribute to parameter estimation. The predictability is defined as the squared Pearson correlation coefficient between the predicted and the observed values of the target trait. Since GA yields different marker subset selections in each iteration, we repeated the 10-fold CV process 10 times and calculated the average predictability for comparison.

## Results

### Performance of the binning method

To demonstrate the suitability of the binning method for dimensional reduction, we compared the predictability of bin genotypes with the PCs using the GBLUP method based on the maize305 dataset and a publicly available maize550 dataset [26]. The maize550 dataset consists of 550 maize hybrids derived from 50 Dent and 41 Flint maize inbred lines. All inbred lines are available in the genomic data, which includes 37 392 high-quality SNP markers. GY and grain dry matter content (GDMC) of the 550 hybrids were measured across four to ten mega-environments in Germany from 1999 to 2014, with genotypes inferred from their parents’ genotypes.

**Figure 2:**
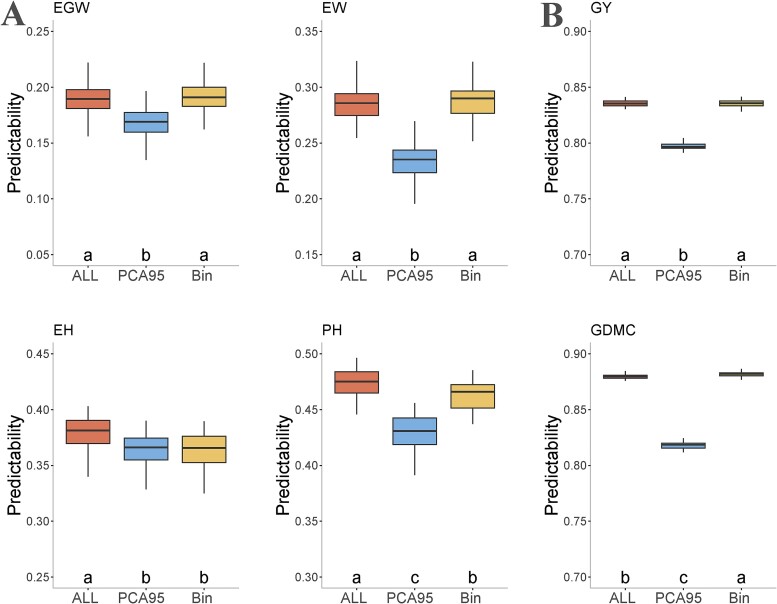
Trait predictability of GBLUP using different dimensional reduction methods on two datasets: maize305 (A) and maize550 (B). The traits include EGW (ear grain weight), EW (ear weight), EH (ear height), PH (plant height), GY (GY), and GDMC (grain dry matter content). The markers are categorized as ALL (all original markers), PCA95 (the top PCs with cumulative explanatory variance of 95%), and Bin (the bin genotype).

For the maize305 and maize550 datasets, a total of 1885 and 1525 bins were generated from the genotype data using the binning algorithm, with the parameter *v* set to 0.15 and 0.1, respectively. Subsequently, 88 PCs and 61 PCs explaining 95% of the total variance of genotypic data (PCA95) were retained, respectively. A 10-fold CV was repeated 50 times to evaluate the predictability of the two dimensionality reduction strategies. For the maize305 dataset, the bin genotypes exhibited significantly higher predictability than the PCA95 genotypes for EW, EGW, and PH, while EH showed comparable performance. Additionally, bin genotyping was equally effective as the conventional method that used all markers for traits EW and EGW (Fig. 2A). In the case of the maize 550 dataset, bin genotypes outperformed PCA95 for all traits, even surpassing the method using all genome-wide markers for trait GDMC (Fig. 2B). Notably, not only does bin genotyping improve predictability but also maintains the positional information of the original SNP markers. This ability of bin genotyping allows us to identify key causal loci underlying target traits, a feature not achievable through PCA. Overall, the results highlight that the binning method is a powerful dimensional reduction strategy. To alleviate the computational burden, bin genotypes were adopted in subsequent studies.

### Predictability of GA–GBLUP

**Figure 3:**
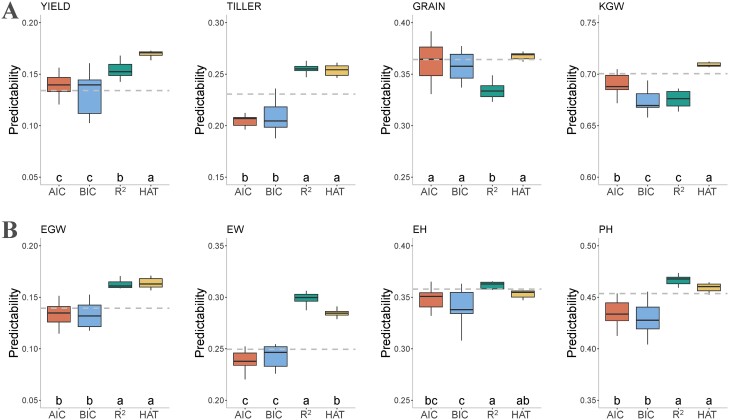
Trait predictability of GA-GBLUP with four different fitness functions on two datasets: rice278 (A) and maize305 (B). The traits include YIELD (grain yield per plant), TILLER (number of tillers per plant), GRAIN (number of grains per panicle), KGW (1000 grain weight), EGW (ear grain weight), EW (ear weight), EH (ear height), and PH (plant height). The fitness functions include AIC, BIC, R^2^, and HAT. The gray dashed line represents the predictability of GBLUP.

We evaluated four GA fitness functions, including AIC, BIC, R^2^, and HAT, and compared the predictabilities of GA-GBLUP and GBLUP using the rice278 and maize305 datasets. In the rice278 dataset, the highest predictability was achieved for KGW (0.6899) across all methods, followed by GRAIN (0.3569), TILLER (0.2310), with the lowest predictability for YIELD (0.1456). Among the four fitness functions, HAT consistently outperformed the other fitness functions, with GA-GBLUP using HAT achieving the highest average predictability (0.3748) across traits, while GA-GBLUP with BIC had the lowest predictability (0.3428) (Fig. 3A). Overall, HAT was superior over AIC, BIC, and R^2^ by 7.35, 9.34, and 5.54%, respectively. The improvement in predictability of GA-GBLUP with the HAT fitness function varied by traits, showing improvements of 26.45% for YIELD, 10.04% for TILLER, 0.60% for GRAIN, and 1.25% for KGW compared to GBLUP.

In the maize305 dataset, PH had the highest predictability (0.4489) across all methods, followed by EH (0.3522), EW (0.2626), and EGW (0.1466). Among the four fitness functions, R^2^ and HAT performed better than AIC and BIC for all four traits when combined with GA-GBLUP, with the average predictability of 0.2894, 0.2857, 0.3225, and 0.3152 for AIC, BIC, R^2^, and HAT, respectively. For EW and EGW, GA-GBLUP with the R^2^ and HAT fitness functions demonstrated superior performance over GBLUP (Fig. 3B). GA-GBLUP with the R^2^ fitness function outperformed GBLUP by 19.84, 16.72, 2.84, and 1.08% for EW, EGW, PH, and EH, respectively. Similarly, the use of the HAT fitness function in GA-GBLUP resulted in 13.8, 17.67, and 1.27% improvements in predictability for EW, EGW, and PH, respectively.

### Marker selection

**Figure 4:**
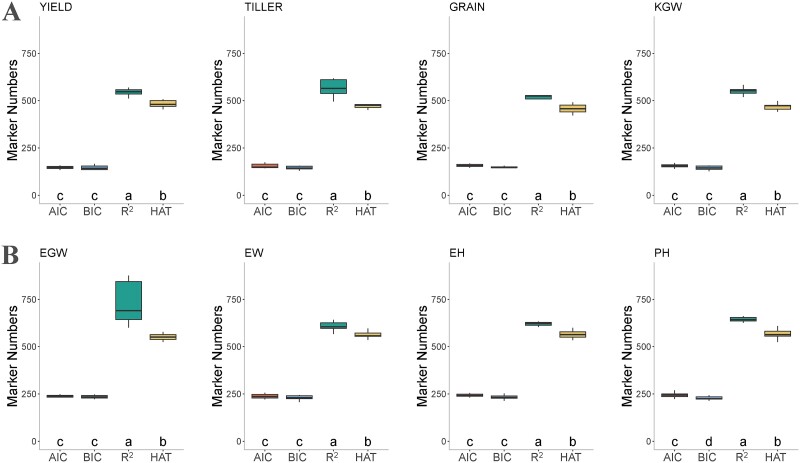
Number of markers selected by GA-GBLUP with four different fitness functions on two datasets: rice278 (A) and maize305 (B). The traits analyzed include YIELD (grain yield per plant), TILLER (number of tillers per plant), GRAIN (number of grains per panicle), KGW (1000 grain weight), EGW (ear grain weight), EW (ear weight), EH (ear height), and PH (plant height). The fitness functions include AIC, BIC, R^2^, and HAT.

To further investigate the superior performance of the fitness functions HAT and R^2^, we examined the number of selected markers by GA-GBLUP equipped with different fitness functions in both the rice and maize datasets. The R^2^ fitness function consistently selected the highest number of markers, followed by the HAT fitness function, with AIC and BIC selecting the lowest number of markers (Fig. 4). In the rice278 dataset, on the average, the percentages of markers selected by AIC, BIC, R^2^, and HAT fitness functions across the four traits were 9.47, 8.99, 34.35, and 29.12%, respectively (Fig. 4A). In the maize dataset, the corresponding percentages were 12.75% for AIC, 12.28% for BIC, 34.55% for R^2^, and 29.82% for HAT (Fig. 4B).

**Figure 5:**
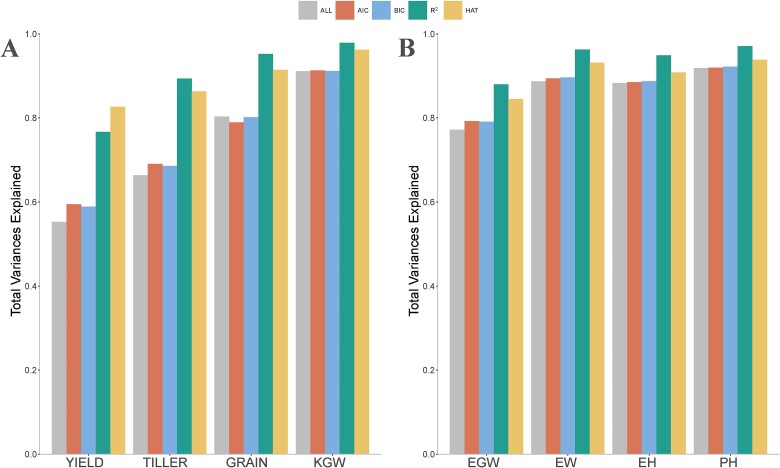
Total variances explained by markers selected with GA-GBLUP under four fitness functions on two datasets: rice278 (A) and maize305 (B). The fitness functions are AIC, BIC, R^2^, and HAT. The scenario labeled ALL represents the total variance explained by all markers.

Finally, we built the kinship matrices using the selected markers and using all markers to estimate the total variances explained by the markers with the REML method. This process was repeated 10 times, with markers selected by GA-GBLUP per run. In the rice278 dataset, KGW had the highest total variance explained by the markers (93.54%) across all methods, while YIELD displayed the lowest total variance explained by the markers (66.59%), which was consistent with their predictability (Fig. 5A). For the four traits, the markers selected by GA-GBLUP with R^2^ and HAT explained 89.79 and 89.16% of the total variance, respectively, surpassing AIC (74.70%) and BIC (74.71%). The total variance explained by the markers selected with GA-GBLUP using R^2^ and HAT was considerably higher than that using all markers (73.27%). In the maize305 dataset, PH demonstrated the highest total variance explained by the markers (93.38%) across all methods. The markers selected by GA-GBLUP with AIC, BIC, R^2^, and HAT explained 87.27, 87.43, 94.06, and 90.58% of the total variance, respectively (Fig. 5B). The variance explained using the kinship matrix built with all markers (86.52%) was much lower than that using markers selected by R^2^ and HAT.

## Discussion

GS has revolutionized hybrid breeding in crops [27–29]. It is well known that accurate prediction is a prerequisite for GS. In this study, we proposed a novel GS method called GA-GBLUP that incorporates the GA. We observed that when R^2^ and HAT fitness functions are used, GA-GBLUP outperforms traditional GBLUP with an increase of predictability up to 26.45%. This amount of improvement is particularly noteworthy for GY, a trait known for its low heritability. According to the breeder’s equation [3], the annual genetic gain is determined by the product of the selection intensity, the genetic variance, and the selection accuracy divided by generation interval. The predictability observed in our study indicates a substantial boost in genetic gain compared with the traditional GS methods.

Among various GS models, GBLUP has been widely applied to animal and plant breeding due to its strong robustness and high computational efficiency [30]. Previous studies have emphasized the significant role of genetic architecture in determining the accuracy of GS [10, 31]. However, GBLUP is often built with the same kinship matrix to predict GEBVs for all traits, regardless of the genetic architectures of the traits. The one-kinship-matrix-fits-all-traits strategy may not fully capture the contributions of trait-specific major-effect markers in GBLUP, potentially leading to lower prediction accuracies [32]. To overcome this problem, researchers have proposed the Blup|GA (Blup-given genetic architecture) approach [33], using publicly available GWAS results to build trait-specific kinship matrices. The results indicate that Blup|GA outperformed GBLUP and BayesB for nine out of eleven traits in a rice diversity dataset. However, the use of inaccurate or inappropriate priors for building the trait-specific kinship matrix may lead to a reduced predictability. Spindel *et al.* [34] proposed a GS + *de novo* GWAS strategy to enhance the predictability, which is effective for certain traits but highly dependent of the identification of highly significant SNPs through the *de novo* GWAS. It is increasingly evident that solely incorporating GWAS signals in GS cannot guarantee improvement in predictability [35].

An alternative approach for identifying trait-specific predictors involves feature selection. Feature selection aims to identify a subset of variables that are mostly related to a target trait, reduce the computational burden and prevent from overfitting [36, 37]. Bayesian methods and the least absolute shrinkage and selection operator (LASSO) [38] are commonly used for selective shrinkage in GS models. These models assume that only a small portion of markers are associated with the target trait. While BayesB may outperform GBLUP in simulated data [1, 39], this is rarely supported in real data analysis [10]. Such a discrepancy may be attributed to the significant impact of priors on parameter estimation for the Bayesian methods [40]. The actual genetic architecture of a trait is often unknown and may differ significantly from the prior assumption. The LASSO method achieves parameter sparsity by applying the L_1_ penalty in regularizing the model. Similar to the priors in the Bayesian methods, however, determining the hyperparameter lambda in the LASSO method can be challenging. An oversized lambda shrinks more coefficients to zero, potentially excluding important markers that are predictive of the target trait, and thus lowers the predictability. Conversely, an undersized lambda provides insufficient regularization and captures noise and irrelevant markers in the training data, resulting in overfitting.

Ever since GA was proposed by Holland in 1975, it has been successfully applied to many areas including operation management, multimedia, wireless networking, and precision agriculture. In the field of bioinformatics, the GA method has been applied to nucleic acid and protein-based sequence analysis, as well as protein structural prediction [41]. However, GA has not yet been explored for GS. Here, for the first time, we used the GA to construct trait-specific kinship matrices to improve the predictability of GBLUP. The advantages of GA-GBLUP include the following: (a) the GA, as a population-based metaheuristic optimization algorithm, can exploit multiple candidate solutions to achieve global or near-global solutions for complex problems. Given the vast solution space in selecting trait-specific predictors (if there are *m* markers in total, there will be 2*^m^* possible solutions), traditional feature selection methods may be trapped in local optima. (b) GA-GBLUP effectively addresses the issue of overfitting commonly encountered in prediction models. Before implementing GA-GBLUP, the original high-dimensional genotype matrix is grouped into bins to reduce complexity. Moreover, GA-GBLUP utilizes a kinship matrix generated from trait-specific markers to capture genetic similarity between individuals, rather than directly fitting the markers. Integrating these approaches makes GA-GBLUP less susceptible to overfitting. The superior performance of GA-GBLUP, as demonstrated in 10-fold CV, provides compelling evidence of its efficacy. (c) GBLUP assumes that predictors are independent and identically distributed (i.i.d.), thus ignoring gene interaction effects that contribute to the quantitative variation of complex traits in plants and animals [42, 43]. GA-GBLUP enables predictors to combine in various ways to determine optimal combinations, potentially considering gene interactions. In addition to GS, GWAS can also be performed using GA-GBLUP to identify bins associated with the target trait.

Among the four fitness functions investigated in our study, the HAT and R^2^ fitness functions exhibit overall superior performance over AIC and BIC. This difference may be attributed to the different emphases of the fitness functions. The number of selected markers and the total variances explained by these markers across different fitness functions may provide some insights. In comparison to GA-GBLUP with the R^2^ and the HAT fitness functions, GA-GBLUP with the AIC and BIC fitness functions selects far fewer markers. Meanwhile, the markers selected by GA-GBLUP with the R^2^ and HAT fitness functions explain a higher total variance than those selected by GA-GBLUP with the AIC and BIC fitness functions. AIC and BIC seek a balance between model complexity and smoothness by including a penalty term on the number of model parameters [22, 23]. However, the penalty term in the AIC and BIC fitness functions may be too severe for GA-GBLUP. Consequently, GA-GBLUP with the AIC and BIC fitness functions tend to be underfitting and the increase in fitness is at the cost of predictability. The HAT fitness function used is a fast algorithm of leave-one-out cross-validation (LOOCV), which helps avoid overfitting by utilizing nearly the entire dataset for training in each iteration, thereby ensuring that the model is robust and generalizes well across all available data. K-fold CV can also be utilized as the fitness function within the GA-GBLUP framework, but the computational time in each iteration would increase about *k* times compared to that of the HAT fitness function. For further improvement of GA-GBLUP, a fitness function with an optimal penalty for marker selection is recommended. Such a fitness function may enable GA-GBLUP to perform both GS and GWAS.

The rapid development of sequencing technologies has facilitated the cost-effective acquisition of large volume biological sequence data, leading to a significant increase in the number of predictors in GS. The surge in predictors poses computational and data analysis challenges due to high dimensionality, referred to as the curse of dimensionality [37]. In such scenarios, GA-GBLUP will slow down significantly due to the exponential growth in the search space and the increased computational complexity for evaluating and optimizing potential solutions. For instance, when evaluating the predictability of a single trait using 10-fold CV in the maize305 dataset, GA-GBLUP with bin genotypes had an average runtime of 205.3 min, whereas GA-GBLUP with original genotypes took about 4067.46 min. This highlights the necessity of implementing an efficient dimensionality reduction method to reduce predictors for GA-GBLUP. PCA is a classical linear dimensionality reduction method widely used in machine learning and data analysis. Nevertheless, PCA may overlook the nonlinear relationships among samples, which can be crucial. Autoencoders are unsupervised deep learning frameworks designed to extract effective nonlinear latent features from unlabeled samples. When effectively trained, latent features obtained via autoencoders work much better than those extracted via PCA [44]. Nonetheless, training autoencoders is time-consuming and requires significant computational resources, posing a challenge for practical application. For genome research, interpretability is desirable. However, similar to the PCs extracted by PCA, interpreting the latent features learned by autoencoders can be challenging. The bin genotypes obtained through the binning method demonstrates superior predictability compared to PCA. Furthermore, the binning method can preserve the positional information of the original markers in the bin genotypes and maintain interpretability, making it an effective tool for dimensionality reduction.

Compared to other GS methods, GA-GBLUP is slow due to numerous generations required for GA to converge towards the optimal solution. The sluggishness of GA is the nature of the method; it is purposely designed to be slow to mimic evolution so that the optimal fitness can be reached globally. Despite the longer processing time of GA-GBLUP, it remains an effective method due to its ability to significantly enhance prediction accuracy. Just as the no-free-lunch theorem states [45], there is no one-size-fits-all solution, and no single algorithm consistently outperforms others, including GA-GBLUP. GA-GBLUP is demonstrated to be effective in predicting traits with low heritability, such as YIELD in the rice dataset, where GA-GBLUP achieved up to 26.45% improvement in predictability. However, when it comes to traits with relatively high heritability, such as KGW in the rice dataset, only a modest 1.25% improvement in predictability is observed when GA-GBLUP is combined with the HAT fitness function. When combined with the R^2^ fitness function, the predictability of GA-GBLUP even drops below that of GBLUP. In general, GA-GBLUP can significantly improve the predictability of traits with low heritability, such as GY, while for traits with relatively high heritability, GBLUP remains the preferred choice.

The newly proposed GA-GBLUP has significantly improved the model predictability. Real data analysis has demonstrated the superiority of GA-GBLUP equipped with the R^2^ and HAT fitness functions compared to the traditional GBLUP method. We recommend GA-GBLUP for traits with low heritability via the new R package GAGBLUP on CRAN for convenient implementation in GS breeding programs. Finally, binning high dimensional genotype data is effective for dimensionality reduction and holds promise for broader applications in interpretable machine learning based GS.

**Key Points**

- We develop a novel GS method named GA-GBLUP, which incorporates the genetic algorithm into the GS method to improve the prediction accuracy.
- GA-GBLUP equipped with the R^2^ and HAT fitness functions demonstrates clear advantages over GBLUP in genomic hybrid breeding, particularly for traits with low heritability.
- The binning method is a superior dimensionality reduction technique in terms of both predictability and interpretability.

## Data Availability

The following datasets can be downloaded from the links below: The rice278 dataset: figshare.com/s/0773080c122d11e58b6306ec4bbcf141 The maize305 dataset: https://doi.org/10.1016/j.cj.2022.09.004 The R code and the software of GA-GBLUP are available at https://CRAN.R-project.org/package=GAGBLUP.
